# Effect of Race on Clinical Outcomes Following Hemodynamically Supported High-Risk Percutaneous Coronary Intervention

**DOI:** 10.1016/j.jscai.2023.100588

**Published:** 2023-03-27

**Authors:** Brittany Fuller, Mir Babar Basir, Cindy L. Grines, Michele Voeltz, Wayne Batchelor, Alexandra J. Lansky, William W. O’Neill

**Affiliations:** a Division of Cardiology, Henry Ford Hospital, Detroit, Michigan; b Northside Hospital Cardiovascular Institute, Atlanta, Georgia; c Inova Heart and Vascular Institute, Falls Church, Virginia; d Division of Cardiology, Yale School of Medicine, New Haven, Connecticut

**Keywords:** health disparities, Impella, mechanical circulatory support, percutaneous coronary intervention, race

Racial disparities persist for non-White populations regarding access, treatment, and outcomes for coronary artery disease.^1^^,^^2^ These may have been relieved, in part, by Medicaid expansion through the Affordable Care Act^3^; however, poor outcomes following percutaneous coronary intervention (PCI) persist, particularly for Black patients. A recent meta-analysis of 10 coronary stent randomized controlled trials found that Black patients had higher rates of diabetes, dyslipidemia, hypertension, and smoking, and a 1.28 hazard ratio for major adverse cardiac events at 5 years compared with White patients (*P* =.01).^2^

## Methods

In a prespecified analysis of the PROTECT II randomized trial (NCT00562016),^4^ we assessed racial differences (non-White vs White patients) in outcomes at 90 days after mechanical circulatory support (MCS) with Impella 2.5 percutaneous left ventricular assist device (pLVAD) compared with intra-aortic balloon pump (IABP) during high-risk PCI (HRPCI). Informed consent was obtained from each participant, and the study conformed to the 1975 Declaration of Helsinki.

A study flow chart is provided in Supplemental Figure S1. Racial categories included White (n = 330) and non-White (n = 95, 63.2% of which identified as Black). Patients concurrently self-identified as both White race and of Hispanic ethnicity were excluded from this analysis (n = 13 patients with pLVAD and n = 10 patients with IABP).

Major adverse events (MAEs), a composite of death, myocardial infarction (MI), stroke/transient ischemic attack, repeat revascularization, cardiac operation, thoracic or abdominal vascular operation, vascular operation for limb ischemia, acute renal dysfunction (need for dialysis, or rise in serum creatinine levels ≥2 times baseline values or >2.5 mg/dL), severe hypotension (defined as systolic blood pressure or augmented diastolic pressure of <90 mm Hg for ≥5 minutes requiring inotropic/pressor medications or IV fluid), cardiopulmonary resuscitation or ventricular arrhythmia requiring cardioversion, increase in aortic insufficiency ≥1 grade, or failure to achieve angiographic success, was assessed at hospital discharge and at 90 days. Major adverse cardiac and cerebrovascular events were defined as all-cause mortality, MI, stroke/transient ischemic attack, or repeat revascularization. Continuous data were compared with unpaired *t* test or Wilcoxon rank sum test and categorical data compared with χ^2^ test. Kaplan–Meier estimates of MAEs to 90 days were compared by log-rank. Statistical analyses were conducted using SAS version 9.4 (SAS Institute).

## Results

**Figure 1:**
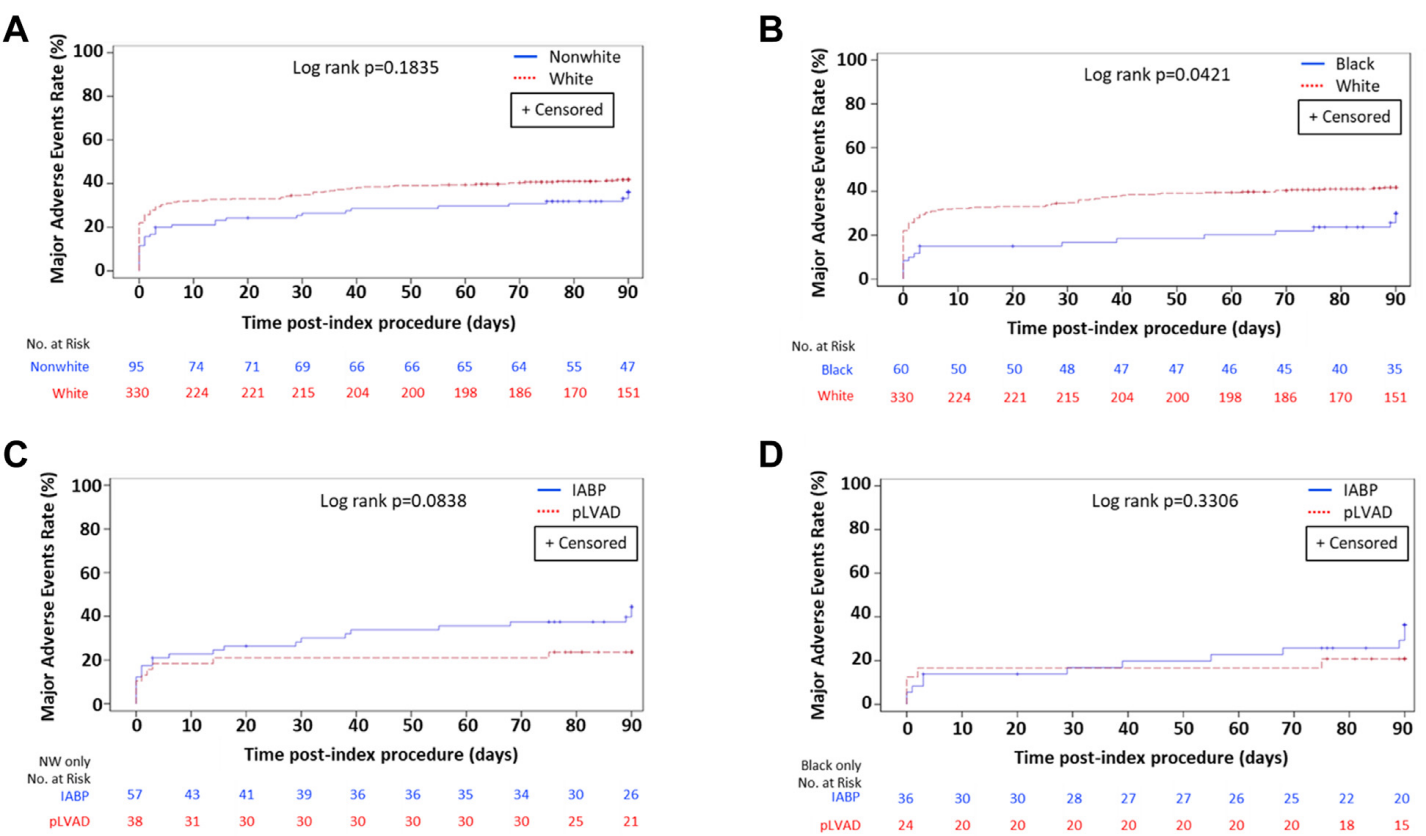
**Kaplan–Meier curve analyses of MAEs to 90 days.** (**A**) Non-White patients compared with White patients (both Impella pLVAD or IABP trial arms), (**B**) Black patients compared with White patients (any trial arm), (**C**) Non-White patients supported with the Impella pLVAD or IABP during HRPCI, and (**D**) Black patients treated with the Impella pLVAD compared with the IABP during HRPCI. HRPCI, high-risk percutaneous coronary intervention; IABP, intra-aortic balloon pulsation; MAE, major adverse event; NW, non-White; pLVAD, percutaneous left ventricular assist device.

Baseline clinical and procedural characteristics in non-White (n = 95) and White patients (n = 330) from the PROTECT II trial are summarized in Supplemental Table S1. Non-White patients were younger, with lower incidence of prior MI, coronary artery bypass graft, and chronic obstructive pulmonary disease, but higher incidence of diabetes. Non-White and White patients had similar SYNTAX scores, number of lesions treated, duration of device support and index procedure, and percent discharged from catheterization laboratory on device support, although atherectomy use was significantly lower in non-White patients (4.2% vs 13.6%; *P* =.011). At 90 days, MAE rates were similar for non-White and White patients (40.9% and 44.5%; *P* =.53) (Supplemental Table S2, Figure 1A) and lower for Black (n = 60) compared with White patients (30.0% and 41.8%; *P* =.042, Figure 1B). However, non-White patients underwent more repeat revascularization (15.1% vs 6.4%; *P* =.008). Non-White patients receiving Impella (n = 38) vs IABP (n = 57) had similar baseline and procedural characteristics, with the exception that non-White patients with pLVAD were less likely to be discharged from the catheterization laboratory on MCS (2.7% vs 40.0%; *P* <.001). MAE rates were lower with pLVAD than that with IABP in non-White patients (26.3% vs 50.9%; *P* =.018) at 90 days (Supplemental Table S2, Figure 1C); major adverse cardiac and cerebrovascular event rates were lower as well (13.2% vs 32.7%; *P* =.032). There was no difference in MAEs for Black patients based on treatment allocation (Figure 1D).

## Discussion

Historically, racial minorities, in particular Black patients, have shown worse clinical outcomes following PCI, with higher mortality, MI, and major adverse cardiac event rates at 1 and 5 years compared with White patients.^1^^,^^2^ Black patients undergoing PCI tend to have more risk factors at a younger age,^1^^,^^2^ and Black patients are also less likely to undergo revascularization (PCI or coronary artery bypass graft) than White patients, regardless of insurance status.^5^

In this context, the findings of our study are as follows: (1) the lack of differences in outcomes between non-White and White patients at 90 days, (2) the better outcomes in Black patients compared with White patients, and (3) the significantly lower MAE rate in non-White patients treated with pLVAD compared with IABP, largely driven by a reduced requirement for repeat revascularization, are noteworthy. In contrast, the higher rate of 90-day repeat revascularization in non-White patients (15% vs 6%) may be due, in part, to less atherectomy use and more diabetes in non-White patients. The use of pLVAD in non-White patients had numerically lower rates of repeat revascularization at 90 days (8% vs 20%) compared with IABP. Whether use of pLVAD allowed for better lesion preparation or hemodynamic improvement, specifically in non-White patients, is possible but speculative.

Although this analysis was prespecified in PROTECT II, randomization was not stratified by race, and there are imbalances in risk factors and procedural characteristics in the groups being compared; therefore, the findings are hypothesis-generating only. PROTECT II was completed in 2012 and represents early experience with Impella, with evolving best practices for HRPCI and MCS strategy in the interim.

## Conclusion

Non-White patients undergoing pLVAD- or IABP-supported HRPCI have similar outcomes compared with White patients at 90 days, and non-White patients treated with pLVAD had significantly better outcomes than those treated with IABP. These findings must be confirmed in a prospective, contemporary trial incorporating current, evidence-based strategies for MCS-supported HRPCI.

## Supplementary material

Analysis Appendix
