# Supplementary material for: Effect of Race on Clinical Outcomes Following Hemodynamically Supported High-Risk Percutaneous Coronary Intervention
Source: J Soc Cardiovasc Angiogr Interv. 2023 Mar 27;2(2):100588. doi: 10.1016/j.jscai.2023.100588 (PMC11307497; doi:10.1016/j.jscai.2023.100588)
Supplement: Analysis Appendix [file mmc1.docx]

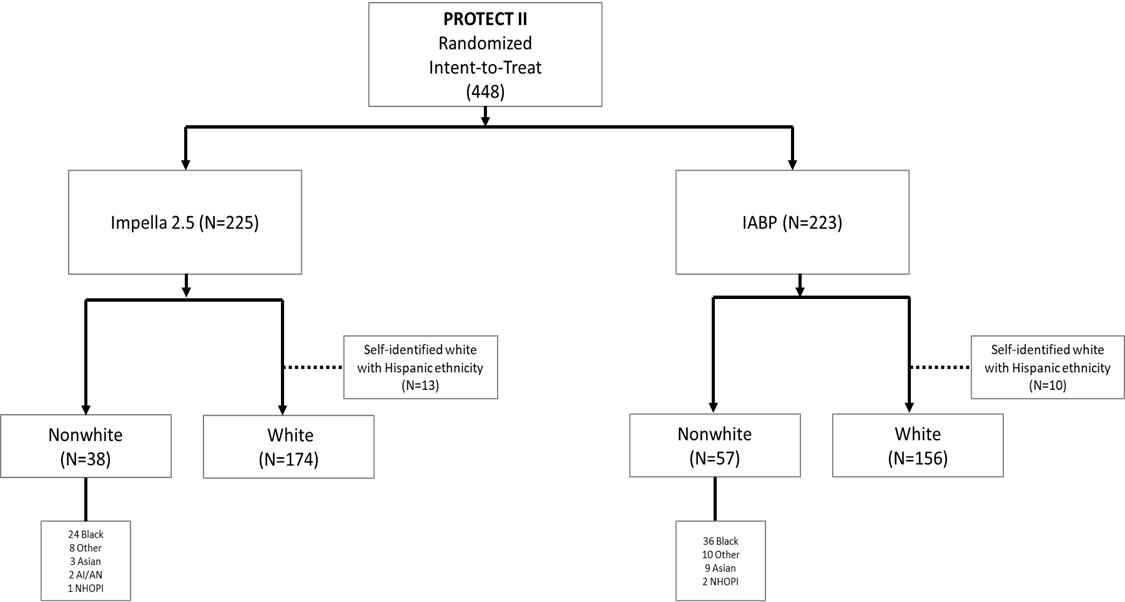


**Supplemental Figure S1.** PROTECT II Study Flow Chart, Race Sub-analysis

AI/AN = American Indian/Alaskan Native; IABP = intra-aortic balloon pump; NHOPI = Native Hawaiian or Other Pacific Islander.

| **Supplemental Table S1.** Baseline characteristics in Non-White and Non-Hispanic White PROTECT II patients | | | | | | |
| --- | --- | --- | --- | --- | --- | --- |
|  | **Non-White**  **(All MCS) (N=95)** | **White**  **(All MCS)  (N=330)** | **P-value** | **Non-White pLVAD  (N=38)** | **Non-White IABP**  **(N=57)** | **P-value** |
| Age (years) | 63.6 ± 10.4 | 68.3 ± 10.6 | <.001 | 64.4 ± 8.4 | 63.0 ± 11.6 | 0.487 |
| Male | 74/95 (77.9) | 268/330 (81.2) | 0.472 | 30/38 (78.9) | 44/57 (77.2) | 0.840 |
| Race |  |  |  |  |  |  |
| AI/AN | 2/95 (2.1) | 0/330 (0.0) | 0.008 | 2/38 (5.3) | 0/57 (0.0) | 0.080 |
| Asian | 12/95 (12.6) | 0/330 (0.0) | <.001 | 3/38 (7.9) | 9/57 (15.8) | 0.256 |
| Black or African American | 60/95 (63.2) | 0/330 (0.0) | <.001 | 24/38 (63.2) | 36/57 (63.2) | 1.000 |
| NHOPI | 3/95 (3.2) | 0/330 (0.0) | 0.001 | 1/38 (2.6) | 2/57 (3.5) | 0.811 |
| Caucasian | 0/95 (0.0) | 330/330 (100 | <.001 | 0/38 (0.0) | 0/57 (0.0) | -- |
| Other | 18/95 (18.9) | 0/330 (0.0) | <.001 | 8/38 (21.1) | 10/57 (17.5) | 0.669 |
| Hispanic/Latino ethnicity | 11/95 (11.6) | -- | -- | 6/38 (15.8) | 5/57 (8.8) | 0.295 |
| LVEF (%) | 23.0 ± 6.1 (95) | 24.3 ± 6.4 (327) | 0.078 | 22.4 ± 5.6 (38) | 23.3 ± 6.5 (57) | 0.467 |
| Prior MI | 52/94 (55.3) | 234/330 (70.9) | 0.004 | 23/37 (62.2) | 29/57 (50.9) | 0.282 |
| NYHA Class III/IV | 48/73 (65.8) | 163/247 (66.0) | 0.970 | 23/31 (74.2) | 25/42 (59.5) | 0.192 |
| Prior PCI | 39/94 (41.5) | 129/329 (39.2) | 0.690 | 18/37 (48.6) | 21/57 (36.8) | 0.256 |
| Prior CABG | 23/95 (24.2) | 120/330 (36.4) | 0.027 | 9/38 (23.7) | 14/57 (24.6) | 0.922 |
| Diabetes mellitus | 57/95 (60.0) | 159/330 (48.2) | 0.042 | 23/38 (60.5) | 34/57 (59.6) | 0.932 |
| Hypertension | 85/95 (89.5) | 281/330 (85.2) | 0.283 | 35/38 (92.1) | 50/57 (87.7) | 0.495 |
| COPD | 16/95 (16.8) | 101/327 (30.9) | 0.007 | 5/38 (13.2) | 11/57 (19.3) | 0.433 |
| Renal insufficiency | 23/95 (24.2) | 92/329 (28.0) | 0.469 | 9/38 (23.7) | 14/57 (24.6) | 0.922 |
| History of tobacco use | 53/94 (56.4) | 243/324 (75.0) | <0.001 | 23/38 (60.5) | 30/56 (53.6) | 0.505 |
| **Admission/procedural characteristics** |  |  |  |  |  |  |
| Additive EuroScore | 7.4 ± 3.4 (95) | 9.0 ± 5.4 (330) | <.001 | 7.7 ± 3.7 (38) | 7.2 ± 3.2 (57) | 0.463 |
| Logistic EuroScore | 14.3 ± 15.3 (95) | 19.6 ± 18.2 (330) | 0.005 | 15.7 ± 18.6 (38) | 13.3 ± 12.8 (57) | 0.476 |
| MAP (mmHg) | 90.2 ± 14.6 (95) | 87.6 ± 15.2 (320) | 0.149 | 92.5 ± 14.0 (38) | 88.6 ± 14.9 (57) | 0.199 |
| Cardiac index (L/min/m^2^) | 2.2 ± 0.6 (71) | 2.3 ± 0.8 (249) | 0.157 | 2.2 ± 0.8 (28) | 2.1 ± 0.5 (43) | 0.630 |
| Number of lesions treated | 3.0 ± 1.5 (95) | 2.8 ± 1.5 (330) | 0.406 | 3.0 ± 1.4 (38) | 3.0 ± 1.5 (57) | 0.954 |
| Use of atherectomy | 4/95 (4.2) | 45/330 (13.6) | 0.011 | 2/38 (5.3) | 2/57 (3.5) | 0.677 |
| Duration of index procedure (hours) | 1.1 ± 0.8 (94) | 1.0 ± 0.6 (327) | 0.755 | 1.1 ± 0.8 (37) | 1.1 ± 0.8 (57) | 0.992 |
| Median duration of device support (hours) | 1.3 (0.8 – 2.6) | 1.35 (0.9 – 2.3) | 0.488* | 1.2 (0.8-1.8; 37) | 1.3 (0.8-6.3; 56) | 0.234* |
| Discharge from cath lab on device support | 23/92 (25.0) | 63/323 (19.5) | 0.251 | 1/37 (2.7) | 22/55 (40.0) | <0.001 |
| SYNTAX Score |  |  |  |  |  |  |
| Pre-PCI | 30.2 ± 14.2 (70) | 29.2 ± 13.1 (214) | 0.586 | 29.2 ± 11.8 (28) | 30.9 ± 15.6 (42) | 0.640 |
| Post-PCI | 14.9 ± 11.8 (70) | 14.6 ± 13.0 (212) | 0.871 | 13.9 ± 10.2 (28) | 15.5 ± 12.8 (42) | 0.594 |
| Change (pre- to post-PCI) | -15.3 ± 10.2 (70) | -14.6 ± 9.2 (212) | 0.584 | -15.3 ± 10.4 (28) | -15.4 ± 10.2 (42) | 0.974 |
| AI, American Indian; AN, Alaska Native; CABG, coronary artery bypass graft; CHF, congestive heart failure; COPD, chronic obstructive pulmonary disease; LVEF, left ventricular ejection fraction; MAP, mean arterial pressure; MI, myocardial infarction; NHOPI, Native Hawaiian or Other Pacific Islander; NYHA, New York Heart Association; PCI, percutaneous coronary intervention; PVD, peripheral vascular disease. Continuous data is reported as mean ± standard deviation; categorical data is reported as percentage.  The 18 patients who self-identified as “Other” race comprised the following, per write-in responses: 5 Mexican, 3 Hispanic, 2 Indian, 1 Arabic, 1 Filipino, 1 Latino, 1 Middle Eastern, 1 Portuguese, 1 Puerto Rican, 1 Russian, 1 South American Indian. | | | | | | |

| **Supplemental Table S2.** CEC-adjudicated MAEs in the Non-White and White PROTECT II patients through 90 days | | | | | | |
| --- | --- | --- | --- | --- | --- | --- |
|  | **Non-White (All MCS) (N=95)** | **White (All MCS)  (N=330)** | **P-value** | **Non-White pLVAD (N=38)** | **Non-White IABP  (N=57)** | **P-value** |
| Composite MAE*, n/N (%) | 38/93 (40.9) | 146/328 (44.5) | 0.531 | 10/38 (26.3) | 28/55 (50.9) | 0.018 |
| Composite MACCE^†^, n/N (%) | 23/93 (24.7) | 83/328 (25.3) | 0.910 | 5/38 (13.2) | 18/55 (32.7) | 0.032 |
| Death, n/N (%) | 6/93 (6.5) | 39/328 (11.9) | 0.134 | 1/38 (2.6) | 5/55 (9.1) | 0.213 |
| MI, n/N (%) | 15/93 (16.1) | 55/328 (16.8) | 0.884 | 4/38 (10.5) | 11/55 (20.0) | 0.222 |
| Q-Wave MI | 1/93 (1.1) | 1/328 (0.3) | 0.340 | 0/38 (0.0) | 1/55 (1.8) | 0.403 |
| Non-Q Wave MI | 14/93 (15.1) | 54/328 (16.5) | 0.744 | 4/38 (10.5) | 10/55 (18.2) | 0.310 |
| CPK-MB ≥3x ULN | 14/93 (15.1) | 54/328 (16.5) | 0.744 | 4/38 (10.5) | 10/55 (18.2) | 0.310 |
| CPK-MB ≥8x ULN | 9/93 (9.7) | 34/328 (10.4) | 0.847 | 3/38 (7.9) | 6/55 (10.9) | 0.629 |
| Stroke/TIA, n/N (%) | 1/93 (1.1) | 7/328 (2.1) | 0.509 | 0/38 (0.0) | 1/55 (1.8) | 0.403 |
| Repeat revascularization (protocol definition), n/N (%) | 14/93 (15.1) | 21/328 (6.4) | 0.008 | 3/38 (7.9) | 11/55 (20.0) | 0.109 |
| Need for cardiac operation, thoracic or abdominal vascular operation, or vascular operation for limb ischemia, n/N (%) | 0/93 (0.0) | 12/328 (3.7) | 0.061 | 0/38 (0.0) | 0/55 (0.0) | -- |
| Acute renal dysfunction^‡^, n/N (%) | 10/93 (10.8) | 31/328 (9.5) | 0.709 | 4/38 (10.5) | 6/55 (10.9) | 0.953 |
| Severe hypotension, n/N (%) | 6/93 (6.5) | 42/328 (12.8) | 0.089 | 1/38 (2.6) | 5/55 (9.1) | 0.213 |
| CPR or ventricular arrhythmia requiring cardioversion, n/N (%) | 8/93 (8.6) | 40/328 (12.2) | 0.336 | 2/38 (5.3) | 6/55 (10.9) | 0.340 |
| Increase in aortic insufficiency > 1 grade^‡§^, n/N (%) | 0/93 (0.0) | 0/328 (0.0) | -- | 0/38 (0.0) | 0/55 (0.0) | -- |
| Failure to achieve angiographic success^¶^, n/N (%) | 2/93 (2.2) | 9/328 (2.7) | 0.752 | 2/38 (5.3) | 0/55 (0.0) | 0.085 |
| CPR, cardiopulmonary resuscitation; MACCE, major adverse cardiac and cerebrovascular events; MAE, major adverse events; TIA, transient ischemic attack; ULN, upper limits of normal. Denominators (for 90-day time point) include those with ≥60 days of follow-up or an event through 90-days post index procedure, unless indicated with §. Event rates reported at 90-days are cumulative from time of index procedure.  ^*^Composite MAE includes all 10 components of the PROTECT II primary endpoint (the 10 individual MAE types listed above). ^†^Retrospectively defined as Major Adverse Cardiac and Cerebrovascular Events with the Myocardial Infarction definition as peak CKMB or troponin values ≥8xULN or other criteria if within 72 hours of PCI and CKMB or troponin values ≥2xULN or other criteria if >72 hours post PCI. ^‡^Events through 120 days (for 90-day time point) were included for two event types (acute renal dysfunction and increase in aortic insufficiency) which were reliant on measurements at the 90-day visit, which had a visit window of ±30 days. ^§^Per echocardiography core laboratory assessment, when available. If not available, site reported data was used. ^¶^Per angiography core laboratory assessment, when available. If not available, site reported data was used. | | | | | | |
